# CoAt-Mixer: Self-attention deep learning framework for left ventricular hypertrophy using electrocardiography

**DOI:** 10.1371/journal.pone.0286916

**Published:** 2023-06-08

**Authors:** Ji Seung Ryu, Solam Lee, Yuseong Chu, Min-Soo Ahn, Young Jun Park, Sejung Yang

**Affiliations:** 1 Department of Precision Medicine, Yonsei University Wonju College of Medicine, Wonju, Korea; 2 Department of Preventive Medicine, Yonsei University Wonju College of Medicine, Wonju, Korea; 3 Department of Dermatology, Yonsei University Wonju College of Medicine, Wonju, Korea; 4 Department of Biomedical Engineering, Yonsei University, Wonju, Korea; 5 Division of Cardiology, Department of Internal Medicine, Wonju Severance Christian Hospital, Yonsei University Wonju College of Medicine, Wonju, Korea; University College Dublin, PAKISTAN

## Abstract

Left ventricular hypertrophy is a significant independent risk factor for all-cause mortality and morbidity, and an accurate diagnosis at an early stage of heart change is clinically significant. Electrocardiography is the most convenient, economical, and non-invasive method for screening in primary care. However, the coincidence rate of the actual left ventricular hypertrophy and diagnostic findings was low, consequently increasing the interest in algorithms using big data and deep learning. We attempted to diagnose left ventricular hypertrophy using big data and deep learning algorithms, and aimed to confirm its diagnostic power according to the differences between males and females. This retrospective study used electrocardiographs obtained at Yonsei University Wonju Severance Christian Hospital, Wonju, Korea, from October 2010 to February 2020. Binary classification was performed for primary screening for left ventricular hypertrophy. Three datasets were used for the experiment: the male, female, and entire dataset. A cutoff for binary classification was defined as the meaningful as a screening test (<132 g/m^2^ vs. ≥132 g/m^2^, <109 g/m^2^ vs. ≥109 g/m^2^). Six types of input were used for the classification tasks. We attempted to determine whether electrocardiography had predictive power for left ventricular hypertrophy diagnosis. For the entire dataset, the model achieved an area under the receiver operating characteristic (AUROC) curve of 0.836 (95% CI, 0.833–838) with a sensitivity of 78.37% (95% CI, 76.79–79.95). For the male dataset, the AUROC was 0.826 (95% CI, 0.822–830) with a sensitivity of 76.73% (95% CI, 75.14–78.33). For the female dataset, the AUROC was 0.772 (95% CI, 0.769–775) with a sensitivity of 72.90% (95% CI, 70.33–75.46). Our model confirmed that left ventricular hypertrophy can be classified to some extent using electrocardiography, demographics, and electrocardiography features. In particular, a learning environment that considered gender differences was constructed. Consequently, the difference in diagnostic power between men and women was confirmed. Our model will help patients with suspected left ventricular hypertrophy to undergo screening tests at a low cost. In addition, our research and attempts will show the expected effect that gender-consideration approaches can help with various currently proposed diagnostic methods.

## Introduction

Left ventricular hypertrophy (LVH) is an increase in left ventricular mass associated with structural alterations of the myocardium. When LVH is present, the risk of developing cardiovascular disease increases by 5–10 times, and similar results have been reported in patients with myocardial infarction [1–4]. Recently, the elderly and obese populations have been increasing due to the average life span extension and the westernized dietary life change, respectively. In general, the number of hypertensive people is increasing. Hypertension is the leading cause of blood vessel overload, resulting in an increased prevalence of LVH [5, 6]. LVH is known to occur in people without the disease; in this case, excessive stress was considered the primary cause. LVH is a risk factor for cardiovascular diseases such as heart failure and arrhythmia. It is closely related to its onset and prognosis [7–9]. Also, LVH is a significant independent risk factor for morbidity and mortality [10, 11]. Therefore, early diagnosis of LVH i.e., early stage of heart change is clinically critical because it can lead to the treatment of patients with cardiovascular disease. Various tests, such as electrocardiography (ECG), echocardiography, computed tomography (CT), and magnetic resonance imaging (MRI), have been used to diagnose LVH. Echocardiography is a relatively accurate method [12–15]. However, the relatively high cost, and need for equipment and specialized health providers are an overburden in primary care.

ECG, a non-invasive test, is widely used for LVH screening because of its shorter test time and lower cost than other tests. An ECG is a diagnostic test that records the microcurrents generated during physiological activities of the body. It has been widely used as a standard test for disease identification, risk stratification, and cardiovascular disease management such as arrhythmias [16–19]. ECG mainly uses the amplitude of the QRS complex as a measure of the classification criteria for screening for LVH [20–23]. However, it is known to be significantly affected by the thickness of the chest wall [24, 25]. To this end, several criteria tables have been proposed, including Sokolow-Lyon and Cornell voltage indexes [26, 27].

However, because it was based only on the voltage size of the QRS complex, the matching rate of actual LVH and diagnostic findings was low. Demographic and anthropometric factors and cardiopulmonary diseases are known to affect the QRS complex [28]. Therefore, it is difficult to apply the proposed criteria to obtain accurate results.

Recently, interest in algorithms using big data and deep learning (DL) has increased. DL algorithms have achieved state-of-the-art performance even for a wide range of classification and identification tasks in the medical field [29, 30]. It was also shown that it is not limited to images and can be applied to various data types. Owing to the applicability of such a DL algorithm, it has also been attempted [31] and applied to bio-signals such as ECG [32, 33]. Initially, it was used for signal processing, such as noise reduction and feature extraction, or short-length ECG, such as the classification of single heartbeats [34–36]. These attempts gradually widened and began to be used for diagnosing cardiovascular diseases and predicting their prognosis [37, 38]. DL algorithms were not limited to one type but were applied in various ways, and showed clinically significant performance in cardiovascular disease diagnosis tasks [39]. As a result, the utilization of DL algorithms in ECG analysis has gained prominence as a promising avenue for specific disease classification. Furthermore, in contrast to conventional rule-based methods, DL algorithms have the capability to uncover ECG features that may have evaded human observation, leading to improved accuracy in diagnosing LVH.

The attention mechanism is used in natural language processing [40]. It is proposed to perform machine translation tasks better and is based on the Recurrent Neural Network (RNN) model. However, in computer vision, Convolutional Neural Networks (CNN) are mainly used as a standard model. Attention in the field of vision is a way to focus on important areas and ignore relatively irrelevant ones. The concept of attention, which mimics a human visual system that seeks to analyze and understand complex scenes efficiently, has inspired researchers to improve performance by applying it to the vision field [41–43]. The attention mechanism in the early vision field was either based on an RNN or was used as a subnetwork [44–46]. Subsequently, it was combined with a CNN module to demonstrate improved performance [47, 48]. In 2017, Google’s transformer first proposed self-attention [49]. Self-attention has shown remarkable performance, and since the introduction of transformers, the attention mechanism in computer vision has gained considerable popularity. Recently, self-attention networks based on transformer networks have emerged, exhibiting great potential [50–52]. These transformer models can replace the existing CNN, and become mainstream architectures in the field of computer vision.

This study attempted to diagnose LVH using big data and DL algorithms. The data consisted of ECGs from 2010 to 2020 at the Yonsei University Wonju Severance Christian Hospital. We designed a CoAt-Mixer, a new model to use self-attention techniques, and analyzed the ECG. In addition, we designed a self-attention-based framework reflecting ECG and demographic features. In addition, differences in LVH diagnosis prediction according to sex were confirmed.

This paper is organized as follows: The background necessary for understanding the paper is summarized in the introduction. The related work was also organized in the introduction. Materials and methods introduce our data collection, overall research overview, and analysis process. The structure of the proposed CoAt-Mixer is also summarized in materials and methods. The results of the model for each dataset and the evaluation environment are detailed in the result section. Grad-CAM, a model analysis method, and results for all metrics are also summarized in the results. The discussion provides an analysis of our result and comparisons against our study and previous study. In addition, it also informs the limitations of our study and the further study to solve them. The conclusion of the study is described in the last paragraph.

## Materials and methods

### Data collection and study approval

**Fig 1:**
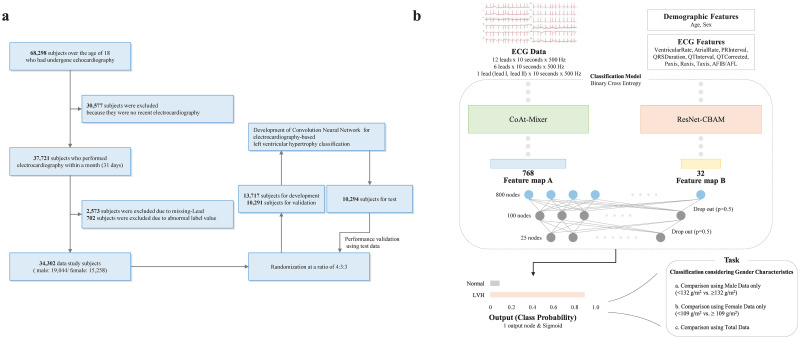
Schematic overview of the study. (a) Flow diagram of patient selection and dataset configuration. (b) Structural diagram of the DL model for LVH classification. Abbreviations: ECG, electrocardiography; LVH, left ventricular hypertrophy.

This retrospective study used ECGs obtained at Yonsei University Wonju Severance Christian Hospital, Wonju, Korea, from October 2010 to February 2020 (Fig 1a). ECGs of subjects aged 18 years or older who visited our hospital during the study period were used. Digital ECGs were recorded using the MUSE Cardiology Information System (GE Healthcare, Chicago, IL, USA). The recorded ECGs were standard supine 10 s 12-lead 500 Hz ECGs, consisting of 5,000 samples. A retrospective chart review collected demographic features, including age and sex, and ECG features, such as ventricular and atrial rates. Echocardiography was performed using an ultrasound scanner with phased-array transducers (Vivid E9, GE Healthcare, Waukesha, Wisconsin). The left ventricular mass index (LVMI) was calculated using the formula recommended by the American Society of Echocardiography [53]. This study was approved by the Institutional Review Board of Yonsei University Wonju Severance Christian Hospital and conformed to the ethical guidelines of the Declaration of Helsinki (approved on January 28, 2021, approval number CR319173). The need for informed consent was waived, given the impracticality and minimal harm.

### Dataset

The dataset consisted of subjects who had undergone both echocardiography and ECG at least once (Fig 1a). The first one was used if the subject has had multiple echocardiography. As a result, only one data was used for each subject. Subsequently, if the selected subject had undergone ECG soon, the dataset was constructed by pairing the recorded ECG and echocardiography diagnosis results. Therefore, for all cases, the difference between the echocardiography and ECG measurements was within a month (31 days). Finally, subjects with abnormal ECG and echocardiography results were excluded. Consequently, 34,302 subjects (19,044 male and 15,258 female subjects) were used as the dataset.

The original data were eight leads (lead I, II, V1, V2, V3, V4, V5, and V6); therefore, four leads (III, aVF, aVR, and aVL) were added using vector calculation, and a total of 12-lead ECG were used for the model. Leads I, II, and III formed a closed circuit using Einthoven’s triangle. Therefore, Lead III was calculated using Kirchhoff’s current law and Lead I and II. The aVR, aVF, and aVL leads, also known as Goldberger leads, consist of the same electrodes in Einthoven’s triangle. Likewise, three leads (aVR, aVF, and aVL) were added using the initial three leads (I, II, and III) and Goldberger’s equation [54]. The ECGs contained a bit of noise generated during the ECG measurement within 1 s before and after. Therefore, 8 s of data were used for model learning. Normalization was not performed because all ECGs were measured at the same resolution. In addition, when the experiment was conducted with ECGs applied to signal processing, such as noise reduction, there was no significant difference between the results of the model. Therefore, the original signal without signal processing was used in this study.

The dataset was partitioned into a training (40%), a validation (30%), and a test set (30%). Due to the large dataset size, utilizing commonly used data ratios may result in significant deviations between sub-datasets. To address this issue and ensure a more realistic clinical application environment, we have expressly set the partition ratio as mentioned above. This ratio aims to minimize the size discrepancies between datasets and facilitate the practical implementation of research findings. Partitioning was performed considering sex (male and female). Each partitioned dataset is combined and used in the DL model to learn the entire dataset. Partitioning was carried out in units of individual subjects, and there were no overlapping subjects between datasets. The training and validation sets were used for model learning and parameter fine-tuning. The model that achieved the highest performance for the validation set was used in the test set, and the final performance of the model was evaluated and verified through it.

### Overall classification framework

Binary classification was performed for primary screening for LVH diagnosis (Fig 1b). Three datasets were used for the experiment: the entire, male, and female dataset. An independent model was used, and in the case of learning with the entire dataset, the label was applied, considering the subject’s gender. Six types of input were used for the classification tasks. We attempted to determine whether ECG had predictive power for LVH diagnosis. This process was repeated by reducing the number of ECG features, demographic features, and ECG leads. Among the 12-lead ECGs, leads I, II, III, aVL, aVR, and aVL were used as 6-lead. Leads I and II were used as the single-lead. Three previously separated datasets were independently used for each task. The models learned using all ECGs and features were selected as the main results. The results of each classification task are summarized in Tables 2 and 3.

To precisely define LVH for binary classification, the LVMI/BSA by the two-dimensional (2D) method was used. LVMI/BSA by the 2D method is the left ventricular mass measured through echocardiography divided by the body surface area. In summary, 132 for males and 109 for females were defined as cut-off values. Moreover, if it is less than the cut-off, it is defined as a subject without LVH, labeled as 0, and vice versa.

### Model architecture and scaling a model

Our framework consists of two models (CoAt-Mixer and ResNet-CBAM) that receive different inputs (ECG and feature data) (Fig 1b). The CoAt-Mixer is designed based on CoAtNet [55] and the Conv-mixer [56]. ResNet-CBAM [48] obtained and modified the prototype model of the original paper, and was able to receive and process one-dimensional (1D) input.

**Fig 2:**
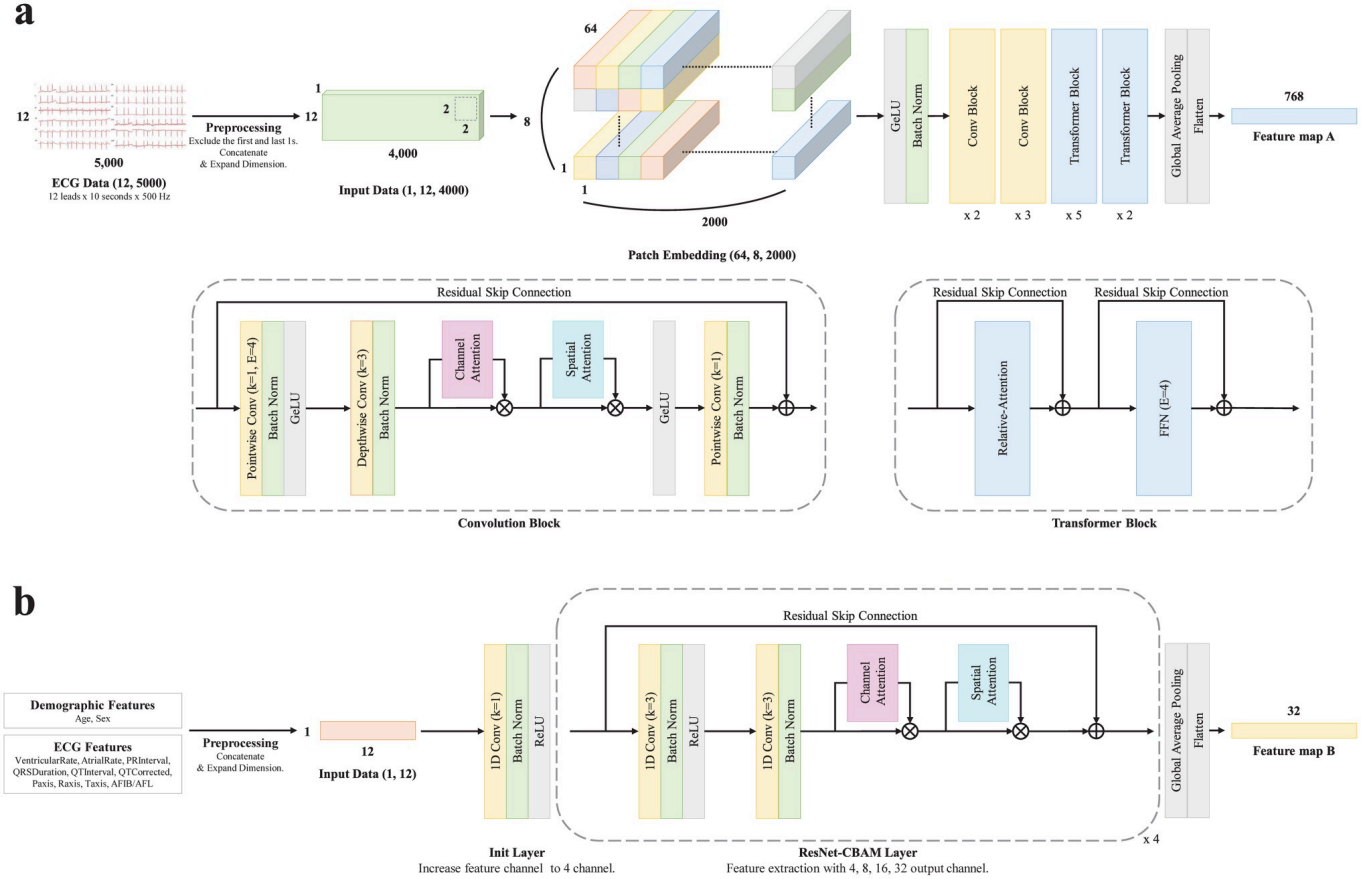
Structural diagram of model. (a) Architectural diagram of the CoAt-Mixer. (b) Architectural diagram of the ResNet-CBAM. Abbreviations: ECG, electrocardiography; GeLU, gaussian error linear unit; FNN, feedforward neural network; ReLU, rectified learning unit.

Our model, called the CoAt-Mixer, is based on C-C-T-T, a vanilla structure presented in the CoAtNet paper (Fig 2a). Previous studies have used a stem layer based on a simple convolution layer. We were inspired by the importance and strength of patch embedding, which the author of the Conv-Mixer paper argued, and used it as the stem layer of the CoAt-Mixer. In patch embedding, patch size of two and embedding dimension of 64 were performed through convolution. In this process, padding size two was added to prevent the size of the vertical axis from decreasing rapidly at the beginning. The convolution block of the CoAt-Mixer comprises an MBConv [57, 58] structure. Here, we transformed the squeeze-excitation (SE) module [47, 58] into a CBAM module to achieve more powerful attention in the convolution process. The transformer block consists of a relative attention [59] and feedforward neural network (FNN). Even without position embedding, sufficient global reactive fields can be obtained with relative attention and patch embedding comprising convolution. 12 (leads) × 8 (seconds) × 500 (Hz) ECG Data were processed and then entered into the CoAt-Mixer in the form of an image (Fig 2a). After passing the patch embedding, the ECG data entered the passed convolution and transformer blocks sequentially. GeLU was used as the activation function. The shape of the feature map past the pooling and flattened layers was 768 × 1.

Two demographic features and 10 ECG features were entered into the ResNet-CBAM model (Fig 2b). The entered feature data increases its channel through the unit layer. Feature data with increased channels pass through the ResNet-CBAM layer, which consists of a convolution layer (kernel size of 3), channel attention module (pooling size of 4), and spatial attention module (pooling size of 12). The ReLU was used as an activation function. The shape of the extracted feature map was 32 × 1.

The features extracted through each model were input to a fully connected layer consisting of three layers (Fig 1b). Each layer had 800, 100, and 25 nodes, and dropout (p = 0.5) was applied simultaneously. The output node of the model is composed of one node, and a sigmoid is used as an activation function. Finally, our designed model outputs predictive probabilities for subjects corresponding to LVH. The binary cross-entropy loss was used as the loss function of the model. The number of subjects with LVH in the training set was relatively small compared to the number of subjects without LHV. Therefore, the relative ratio was calculated and applied to the loss function to supplement the learning process. The hyperparameters were determined through repetitive experiments and grid searches. The batch size, learning rate, number of each block in CoAt-Mixer, channel size, and ResNet-CBAM layer count were considered potential hyperparameters. The learning rate was between 0.0001 and 0.1, and the batch size was between 8 and 512. The number of blocks, layers, and channel size of the model were determined by referring to previous studies.

### Primary outcome and statistical metrics

Continuous variables are presented as mean and standard deviation (SD). To organize the dataset’s variables, independence, normality, and equal variance were tested. First, in partitioning the dataset, we proceeded with partitioning according to individual patient units. Therefore, each dataset (male without LHV vs. male with LVH, female without LHV vs. female with LVH) was assumed to be independent. The Kolmogorov-Smirnov test and D’agostino-Pearson test were used to determine whether each group had normality. A male with LVH, which had a relatively small number of samples, was tested using the Shapiro-Wilk test. As a result, the null hypothesis was adopted, with a significance probability of less than 0.001 for all variables. As a result of the previous test, it was confirmed that all variables had normality; therefore, the equally distributed test was performed with the Bartlett test. Based on the equal variance test results, an independent two-sample t-test was used to determine whether there was a significant difference between groups. The test results are presented in Table 1.

**Table 1:** Dataset characteristics.

	Male			Female		
	Without LVH	LVH	P-value	Without LVH	LVH	P-value
No. of unique subjects	17,811	1,233		11,618	3,640	
**Demographic features**						
Age (years), mean (SD)	61.61 (14.96)	67.72 (13.93)	< 0.001	63.38 (15.78)	72.26 (12.30)	< 0.001
**Echocardiography features**						
LV EF	62.26 (10.29)	56.97 (14.60)	< 0.001	64.55 (7.67)	62.00 (11.62)	< 0.001
LV dimension diastolic	5.22 (1.05)	5.88 (1.74)	< 0.001	4.87 (0.81)	5.31 (0.49)	< 0.001
LV dimension systolic	3.54 (6.19)	4.06 (0.98)	0.003	3.16 (0.93)	3.53 (1.19)	< 0.001
Ventricular septum thickness diastolic, mean (SD)	0.85 (0.17)	1.06 (0.20)	< 0.001	0.79 (0.10)	0.96 (0.14)	< 0.001
Ventricular septum thickness systolic, mean (SD)	1.17 (0.66)	1.35 (0.27)	< 0.001	1.12 (1.44)	1.26 (0.30)	< 0.001
Posterior wall thickness diastolic, mean (SD)	0.86 (0.16)	1.05 (0.29)	< 0.001	0.80 (0.13)	0.96 (0.17)	< 0.001
Posterior wall thickness systolic, mean (SD)	1.24 (0.73)	1.37 (0.25)	< 0.001	1.17 (0.25)	1.32 (0.21)	< 0.001
LV mass index/BSA, mean (SD)	90.75 (16.93)	146.86 (12.23)	< 0.001	83.93 (13.88)	126.96 (15.54)	< 0.001
LA volume index, mean (SD)	30.49 (12.29)	43.20 (20.52)	< 0.001	30.79 (12.00)	40.94 (17.95)	< 0.001
**Electrocardiography features**						
Ventricular rate, mean (SD)	74.05 (16.71)	74.47 (17.45)	0.420	75.26 (16.00)	74.94 (16.44)	0.304
Atrial rate, mean (SD)	74.14 (17.16)	74.53 (17.79)	0.446	75.33 (16.42)	74.97 (16.56)	0.249
PR interval, mean (SD)	164.62 (27.38)	167.79 (29.78)	< 0.001	159.28 (25.83)	163.39 (26.99)	< 0.001
QRS duration, mean (SD)	95.72 (15.64)	101.17 (19.01)	< 0.001	87.41 (13.54)	91.53 (16.74)	< 0.001
QT interval, mean (SD)	396.86 (39.49)	414.22 (44.23)	< 0.001	400.87 (40.82)	412.80 (46.56)	< 0.001
QT corrected, mean (SD)	434.03 (34.38)	453.63 (37.81)	< 0.001	442.28 (32.80)	453.87 (37.93)	< 0.001
P axis, mean (SD)	50.54 (23.00)	47.19 (25.43)	< 0.001	46.07 (22.96)	43.87 (23.88)	< 0.001
R axis, mean (SD)	34.46 (42.54)	23.83 (43.57)	< 0.001	36.12 (35.80)	24.16 (35.57)	< 0.001
T axis, mean (SD)	45.64 (39.81)	68.35 (67.06)	< 0.001	42.25 (38.33)	56.68 (57.30)	< 0.001

Abbreviations: LVH, left ventricular hypertrophy; SD, standard deviation; LV, left ventricular; LA, left atrium; EF, ejection fraction; BSA, body surface area

**Fig 3:**
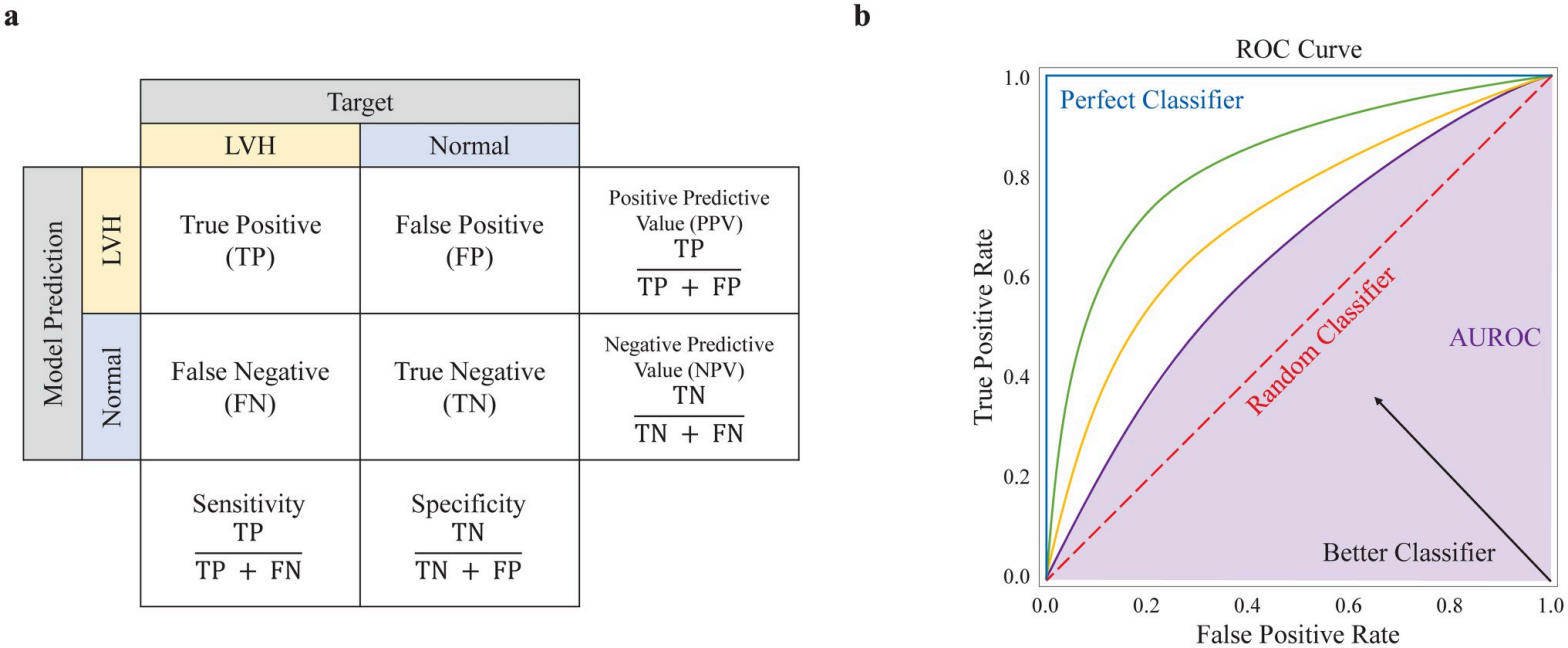
Visualization of statistical metrics. (a) Confusion matrix and statistical metrics for binary classification. (b) ROC curve and AUROC. Abbreviations: LVH, left ventricular hypertrophy; ROC, receiver operating characteristic; AUROC, area under receiver operating characteristics curve.

In this study, we considered the predictive power of the LVH model for LVH as the primary outcome. The area under the receiver operating characteristic curve (AUROC) was used as a significant indicator for the model performance evaluation (Fig 3b). Other statistical metrics included sensitivity, specificity, positive predictive value (PPV), negative predictive value (NPV), and F1 score. According to the classification results of the model and comparison between actual labels, ECGs were subdivided into true positive (TP), false negative (FN), true negative (TN), and false positive (FP). This was then used to calculate the metrics (Fig 3a). The optimal cutoff with the receiver operating characteristic (ROC) curve to minimize the misclassification of the model was calculated based on the maximum Youden index (J). The supplementary material summarizes the accuracy, sensitivity, specificity, PPV, NPV, and F1 score measured using the optimal cutoff. The metrics are calculated as follows:

$$F1\;score=2\times \frac{\left(Sensitivity\;\times \;PPV\right)}{\left(Sensitivity\;+\;PPV\right)}$$ (1)

$$J=Max_{C}\left(Sensitivity+\;Specificity-1\right),$$ (2)

where C is the cut-off point in the ROC curve.

The Yonden index was calculated using the validation set, and all outcomes were measured for the test dataset. All statistics data were reported as point estimates and 95% confidence intervals (CIs). Data were analyzed and visualized using Python 3.9.5 (Python Software Foundation).

### Visualization for model explanation

We used gradient-weighted class activation (Grad-CAM) [60] to visualize the prediction of the model. Grad-CAM is a model analysis methodology that is applicable to CNN models. This is a representative method for visualizing the decision of the CNN model. The ECGs used were 1D inputs. Therefore, Grad-CAM was approximated by a 2D plane and converted into an image. Just before global average pooling, the size of the heat map extracted from our model was a one-line array (1 × 125). Using resizing and interpolation, the heat map was resized to fit the size of the ECG image. A Grad-CAM was constructed by combining the ECG image and the adjusted heat map. The red area of Grad-CAM refers to the part the DL model viewed as necessary in making the final decision. Additionally, the closer this area is to red, the more meaningful the interpretation can be. Conversely, the blue and index areas, similar to the blue series, can be viewed as being relatively less important.

## Results

### Study population

A total of 34,302 subjects were included in this study (Fig 1a). Of the subjects, 19,044 (55.51%) were male, and 1,233 subjects had LVH. The average age of male subjects with LVH is 67.72 (SD 13.93), and the average LV mass index/BSA value is 146.86 (SD 12.23) (Table 1). As a result of statistical analysis between the subject groups that did not have LVH, it was confirmed that there was a significant difference between the two groups (p<0.001). Conversely, 15,258 (44.48) subjects were female, and 3,640 had LVH. The average age of female subjects with LVH is 72.26 (SD 12.30), and the average LV mass index/BSA value is 126.96 (SD 15.54). As in the case of male subjects, it was confirmed that there was a significant difference between the two groups (p<0.001). Ejection fraction was lower in LVH group, and LV dimension and thickness were higher in LVH group compare with without LVH group. PR interval, QRS duration, QTc was higher in LVH group compare without LVH group.

### Evaluation protocol

The ECGs signals used in this experiment were measured using devices developed by the same company. The number of volts per A/D bit was 4.88, the amplitude unit at microvolts, and the A/D converter at 16-bit resolution. The resolution was the same, because it was set in the same setup environment during the experimental period. In addition, when the experiment was conducted with signal-treated ECGs, such as noise reduction or normalization, no significant difference was observed between the experimental results. Therefore, to preserve the morphology of the original signal, we used raw ECGs. The optimal batch size was 32, and the initial learning rate was 0.001. Two and three convolution blocks and five and two transformer blocks of CoAt-Mixer and were used, respectively. The output channels of each block were set as 96, 192, 384, and 768. The ResNet-CBAM layer was set to 4. However, the difference between the model results based on the hyperparameter set was small. Models were developed for 100 epochs using the Adam Optimizer. During the learning process, the epoch with the highest AUROC for the validation set was defined as the optimal state. Subsequently, the final performance was evaluated and verified along with the optimal model and test set. PyTorch version 1.7.1 and NVIDIA GeForce RTX 2080 Ti were used as the DL framework.

### LVH estimation

**Fig 4:**
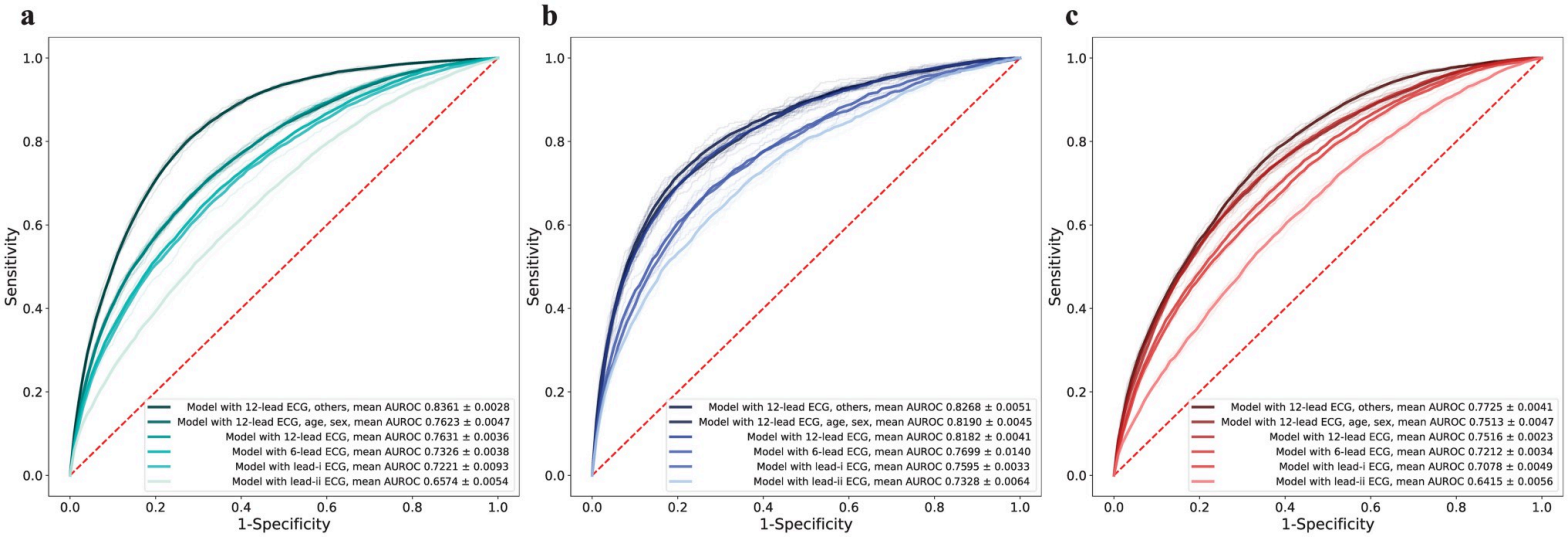
Performance of LVH prediction model for gender. (a) Receiver operating characteristic curve with 95% CI for predicting LVH in entire dataset. (b) Receiver operating characteristic curve with 95% CI for predicting LVH in male dataset. (c) Receiver operating characteristic curve with 95% CI for predicting LVH in female dataset. Abbreviations: AUROC, area under receiver operating characteristics curve; LVH, left ventricular hypertrophy; ECG, electrocardiography; CI, confidence interval.

A performance summary of the model is provided in Fig 4 and Tables 2 and 3. S1 Table provides a performance summary using 10-fold cross validation.

**Table 2:** Performance metrics for classification task with Youden index.

Gender	Input Data	Metrics					
		AUROC (95% CI)	Sensitivity (95% CI)	Specificity (95% CI)	PPV(95% CI)	NPV (95% CI)	F1 score (95% CI)
Entire	12-Lead ECGs Demographics, ECG features	0.836 (0.833–0.838)	78.37% (76.79–79.95)	74.04% (72.70–75.38)	33.41% (32.61–34.21)	95.38% (95.14–95.63)	46.85% (45.78–47.92)
	12-Lead ECGs Demographics features	0.762 (0.758–0.765)	69.62% (67.75–71.49)	68.74% (66.58–70.911)	27.05% (26.25–27.84)	93.18% (92.97–93.39)	38.96% (37.84–40.08)
	12-Lead ECGs	0.763 (0.760–0.765)	68.04% (65.79–70.29)	70.48% (68.41–72.54)	27.72% (26.97–28.47)	93.02% (92.74–93.30)	39.39% (38.26–40.53)
	6-Lead ECGs	0.732 (0.727–0.737)	63.07% (56.23–69.91)	70.19% (64.92–75.46)	26.08% (24.67–27.49)	92.01% (91.19–92.82)	36.90% (34.29–39.46)
Male	12-Lead ECGs Demographics, ECG features	0.826 (0.822–0.830)	76.73% (75.14–78.33)	74.42% (72.52–76.33)	17.33% (16.50–18.15)	97.87% (97.77–97.98)	28.27% (27.06–29.48)
	12-Lead ECGs Demographics features	0.819 (0.815–0.822)	72.37% (68.29–76.44)	76.21% (71.38–81.04)	18.10% (15.94–20.26)	97.56% (97.36–97.77)	28.96% (25.85–32.03)
	12-Lead ECGs	0.818 (0.815–0.821)	73.66% (71.56–75.76)	75.23% (72.64–77.82)	17.27% (16.24–18.31)	97.63% (97.52–97.74)	27.99% (26.48–29.49)
	6-Lead ECGs	0.769 (0.750–0.789)	67.70% (61.20–74.21)	73.06% (68.84–77.29)	14.93% (13.66–16.20)	97.03% (96.56–97.50)	24.46% (22.33–26.59)
Female	12-Lead ECGs Demographics, ECG features	0.772 (0.769–0.775)	72.90% (70.33–75.46)	66.81% (64.21–69.41)	40.89% (39.80–41.99)	88.76% (88.18–89.33)	52.40% (50.83–53.96)
	12-Lead ECGs Demographics features	0.751 (0.747–0.754)	70.41% (68.07–72.74)	66.31% (64.18–68.44)	39.65% (38.78–40.52)	87.75% (87.17–88.33)	50.73% (49.41–52.05)
	12-Lead ECGs	0.751 (0.749–0.753)	70.01% (68.13–71.90)	67.33% (65.23–69.44)	40.26% (39.41–41.12)	87.77% (87.40–88.13)	51.13% (49.93–52.32)
	6-Lead ECGs	0.721 (0.716–0.725)	69.33% (63.19–75.47)	62.05% (55.50–68.60)	36.57% (34.40–38.74)	86.64% (85.52–87.77)	47.88% (44.55–51.20)

Abbreviations: AUROC, area under the receiver operating characteristic curve; CI, confidence interval; PPV, positive predictive value; NPV, negative predictive value

**Table 3:** Performance metrics for single-lead electrocardiography (lead I, II excerpt) with Youden index.

Gender	Input Data	Metrics					
		AUROC (95% CI)	Sensitivity (95% CI)	Specificity (95% CI)	PPV (95% CI)	NPV (95% CI)	F1 score (95% CI)
Entire	Lead I	0.722 (0.709–0.735)	60.16% (56.83–63.48)	71.45% (68.77–74.13)	25.93% (24.87–26.98)	91.54% (91.12–91.96)	36.24% (34.60–37.87)
	Lead II	0.657 (0.649–0.664)	59.04% (52.81–65.26)	62.75% (55.87–69.64)	20.93% (19.38–22.49)	90.25% (89.71–90.79)	30.91% (28.36–33.45)
Male	Lead I	0.759 (0.754–0.764)	70.40% (64.63–76.17)	69.11% (62.72–75.50)	13.80% (12.35–15.24)	97.12% (96.81–97.43)	23.08% (20.75–25.40)
	Lead II	0.732 (0.723–0.741)	63.45% (58.12–68.77)	70.75% (66.63–74.86)	13.14% (12.24–14.04)	96.54% (96.23–96.85)	21.77% (20.23–23.31)
Female	Lead I	0.707 (0.700–0.714)	64.02% (59.85–68.19)	64.72% (61.00–68.45)	36.31% (35.18–37.44)	85.18% (84.36–86.00)	46.34% (44.31–48.34)
	Lead II	0.641 (0.633–0.649)	61.15% (49.73–72.56)	58.45% (47.30–69.61)	31.81% (29.88–33.75)	82.91% (81.37–84.45)	41.85% (37.33–46.07)

Abbreviations: AUROC, area under the receiver operating characteristic curve; CI, confidence interval; PPV, positive predictive value; NPV, negative predictive value

For the entire dataset, the model with the highest performance in the validation set showed an AUROC curve of 0.836 (95% CI, 0.833–838) in the test set. For other metrics, the mean sensitivity was 78.37% (95% CI, 76.79%–79.95%), and the mean specificity was 74.04% (95% CI, 72.70%–75.38%) (Table 2). The model with 12-lead ECG and sex, age, and 12-lead ECG alone showed AUROC curves of 0.762 (95% CI, 0.758–765) and 0.763 (95% CI, 0.760–765), respectively. The AUROC of the model with a 6-lead ECG was 0.732 (95% CI, 0.727–737), which showed a slight decrease in performance when the augmented limb lead (lead V1, V2, V3, V4, V5, and V6) disappeared. The models with single-lead ECG showed inferior performance [AUROC, 0.722 (95% CI, 0.709–0.735) and 0.657 (95% CI, 0.649–0.664), respectively]. The model using all inputs showed a high diagnostic ability when using the entire dataset. However, because the ECG features were excluded from the input, the performance rapidly decreased.

The model with the male dataset showed the expected classification performance [AUROC, 0.826 (95% CI, 0.822–0.830)], similar to the entire dataset [AUROC, 0.836 (95% CI, 0.833–838)]. For other metrics, the mean sensitivity was 76.73% (95% CI, 75.14%–78.33%) and the mean specificity was 74.42% (95% CI, 72.52%–76.33%) (Table 2). No significant difference was observed between the models in which a 12-lead ECG was included in the input [AUROC, 0.819 (95% CI, 0.815–0.822), 0.818 (95% CI, 0.815–0.821)]. The 6-lead and single-lead models showed low performance. [AUROC, 0.769 (95% CI, 0.750–0.789), 0.759 (95% CI, 0.754–0.764), and 0.732 (95% CI, 0.723–0.741)]. Unlike the models with the entire dataset, models with the male dataset showed different trends. A certain level of performance was maintained even if the demographics or ECG features were omitted from the input.

For the female dataset, the model with the highest performance in the validation set showed an AUROC curve of 0.772 (95% CI: 0.769–775) in the test set. For other metrics, the mean sensitivity was 72.90% (95% CI, 70.33%–75.46%) and the mean specificity was 68.81% (95% CI, 64.21%–69.41%) (Table 2). Other models with female data showed unsatisfactory overall performance regardless of the input [AUROC, 751 (95% CI, 0.747–0.754), 0.751 (95% CI, 0.749–0.753), 0.721 (95% CI, 0.716–0.725)]. The models with single-lead ECG had no diagnostic power [AUROC, 0.707 (95% CI, 0.700–0.714), 0.641 (95% CI, 0.633–0.649)].

### Visualization for explainable AI

**Fig 5:**
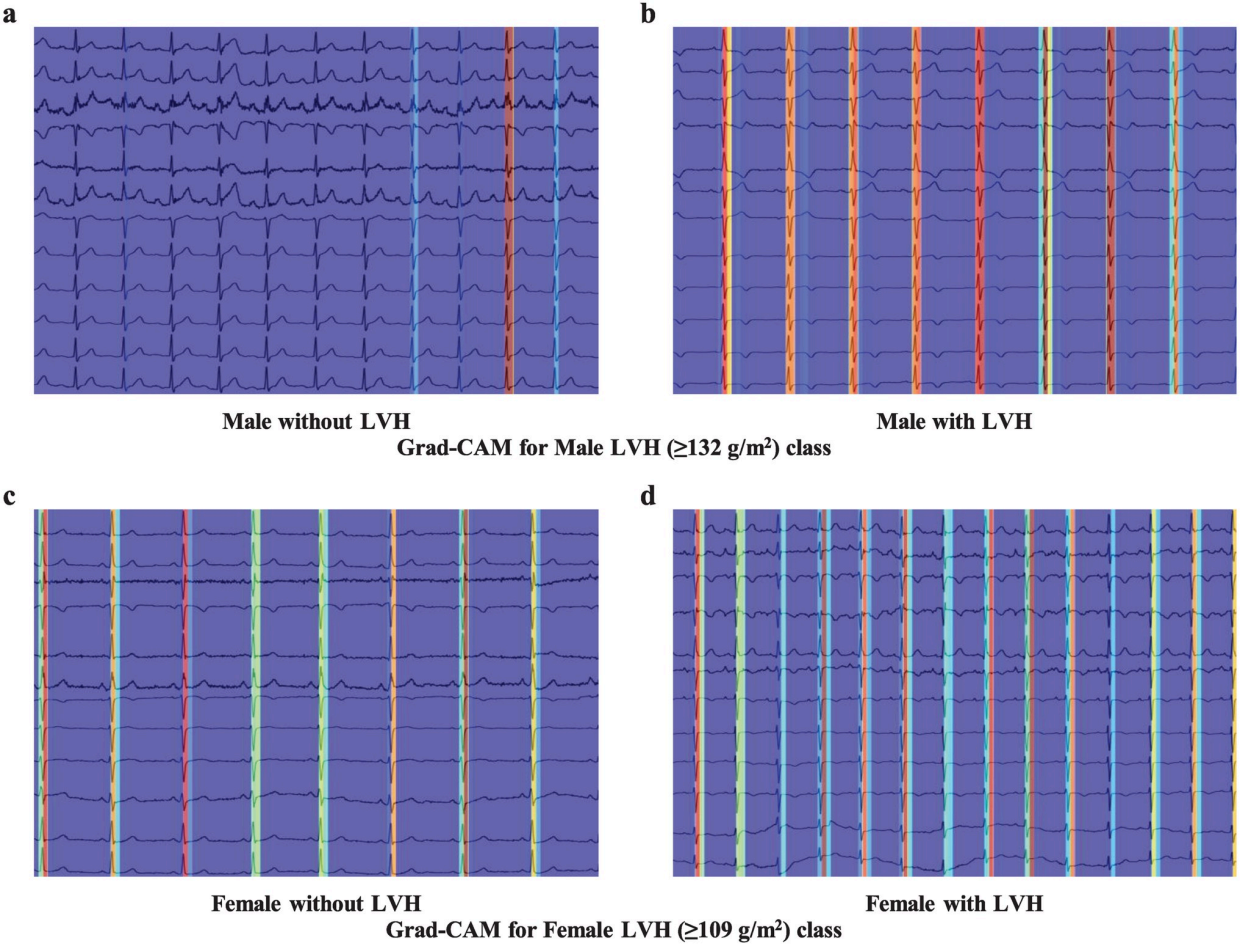
Class activation maps. (a, b) Grad-CAM for male with LVH class (≥132 g/m^2^). The ECGs of male subjects without LVH (<132 g/m2) in panel (a) attracted minimal attention from the model predicting LVH. On the other hand, the model showed strong attention to ECGs with LVH. (c, d) Grad-CAM for female with LVH class (≥109 g/m^2^). The ECGs of female subjects without LVH (<109 g/m2) in panel (c) attracted minimal attention from the model predicting LVH. On the other hand, the model showed strong attention to ECGs with LVH. Abbreviations: Grad-CAM, gradient-weighted class activation map; ECG, electrocardiography; LVH, left ventricular hypertrophy.

Fig 5 summarizes the Grad-CAM results for subjects without LVH and those with LVH according to sex. For all classes, activation for LVH was visualized. For male subjects with LVH (Fig 5b), the model showed intense activation of the QRS complex. For male subjects without LVH (Fig 5a), the model showed the same composition, but low activation. For ECGs obtained from female subjects with and without LVH, the model tended to focus on the QRS complex. The model showed strong activation in female subjects with LVH during low activation in the opposite class.

## Discussion

Our study confirmed whether DL algorithms can diagnose LVH using ECGs, demographics, and ECG features. To consider the difference in male and female, we separated the dataset, confirmed the results, and confirmed that there was a difference in LVH diagnosis. Previous studies have reported differences in heart characteristics between subjects with and without LVH. Subjects with LVH mainly had a low EF [61] and a relatively long PR interval and QRS duration [62, 63]. The reason for this result is that cardiac remodeling occurs because of LVH, and remodeling causes an increase in the ratio of wall thickness and an increase in chamber dimension [61]. These characteristics were confirmed in our dataset (Table 1), and the artificial intelligence model could be used for diagnosis. Our findings confirmed that the model showed high sensitivity (78.37%, 76.73%, and 72.90%) (Table 2) and could diagnose LVH.

Other studies have reported that sex affects the ECG. It has been reported that there are differences in QT duration, J-ST voltage, and T wave form depending on sex hormones such as testosterone. The ECG of females was found to have shorter P wave, shorter PR intervals, lower and narrower QRS complexes, longer QT/JT intervals, lower J-points, ST-segment amplitudes, and lower and broader T waves [64]. In addition, it has been reported that male hormones secreted during adolescence affect LV wall thickness and heart rate [65]. Thus, Males have greater QRS amplitudes and longer QRS durations than females. As a result, these factors suggest that it is relatively difficult to reduce changes in ECG compared to males. These sex differences are not limited to morphological differences in ECG. It has been reported that there is a difference in the prevalence of ECG abnormalities such as sinus bradycardia, bundle branch block, ST-T wave abnormalities, and LVH between males and females [66]. In addition, it has been reported that females have low accuracy for Sokolow-Lyon and Cornell voltage indexes owing to differences between QRS duration and amplitude [67, 68]. In our study, the model that learned only male data had an AUROC of 0.826, which showed better predictive power than that of females (AUROC 0.772). We combined demographics and ECG features with an ECG so that the model can learn by considering gender differences. However, in the diagnosis of LVH, it was not possible to increase the predictive power of female sex to the same extent as that of male sex. This low predictive power may be too complex for the model to capture the characteristics for classification because the changes in QRS amplitude and duration are relatively insignificant. In addition, sex differences in LV mass index/BSA used as labels may not fully reflect sex differences in QRS duration and voltage.

Prior to this study, several studies were conducted to diagnose LVH using machine learning (ML) and DL algorithms (Table 4). Research using the DL algorithm was recently conducted by Joon-Myoung Kwon et al. [69]. It is the first study using large-scale ECG datasets and artificial intelligence. ECG and demographic/biometric information were used. However, in terms of diagnosing LVH, biased results were shown, and the sensitivity was lower than that of the traditional methods. In addition, there seemed to be room for performance improvement [70]. CoAt-Mixer shows that the AUROC and specificity are slightly lower than those in previous studies using DL. However, CoAt-Mixer showed a better search for subjects with LVH, even though the class imbalance was higher [sensitivity, 78.37% (95% CI, 76.79–79.95) vs. 49.6% (95% CI, 49.0–50.2)]. From the results of the research using ML algorithms and this study, it is difficult to determine whether it is superior because there is a difference between the number of subjects and the class distribution. This study used a dataset comprising broader age groups and more subjects. It shows a better generalization performance than Fernando De la Garza Salazar et al. did [71]. Another study, Lim Yan Zheng Daniel et al. [72], was accompanied by a large number of subjects, up to 17,310. However, the subjects were limited to men aged between 16 and 23 years. In this study, more clinical effects can be expected because it consisted of an age group with a high frequency of risk from LVH.

**Table 4:** Comparison with previous studies.

	Methods	Development Data (LVH/ without LVH)	Test Data (LVH/ without LVH)	AUROC (95% CI)	Sensitivity (95% CI)	Specificity (95% CI)	PPV (95% CI)	NPV (95% CI)
**Ours**	Model with Entire dataset	24,008 (3,409/ 20,599)	10,294 (1,464/ 8,830)	0.836 (0.833–0.838)	78.37% (76.79–79.95)	74.04% (72.70–75.38)	33.41% (32.61–34.21)	95.38% (95.14–95.63)
	Model with Male dataset	13,329 (862/ 12,467)	5,715 (371/ 5,344)	0.826 (0.822–0.830)	76.73% (75.14–78.33)	74.42% (72.52–76.33)	17.33% (16.50–18.15)	97.87% (97.77–97.98)
	Model with Female dataset	10,679 (2,547/ 8,132)	4,579 (1,093/ 3,486)	0.772 (0.769–0.775)	72.90% (70.33–75.46)	66.81% (64.21–69.41)	40.89% (39.80–41.99)	88.76% (88.18–89.33)
**Joon-Myoung Kwon. et al**. [69]	Ensemble Neural Network	12,648 (2,794/ 9,854)	3,162 (698/ 2,464)	0.880 (0.877–0.883)	–	–	–	–
	Ensemble Neural Network*	12,648 (2,794/ 9,854)	5,479 (861/ 4,615)	0.868 (0.865–0.871)	49.6% (49.0–50.2)	93.6% (93.4–93.7)	–	–
	Convolutional Neural Network*	12,648 (2,794/ 9,854)	5,479 (861/ 4,615)	0.854 (0.850–0.858)	43.8% (43.1–44.6)	93.6% (93.3–93.7)	–	–
**De la Garza Salazar, F. et al**. [71]	CHCM	307 (142/ 165)	132 (61/ 71)	–	74.3%	68.7%	53.8%	84.5%
	CHCM*	307 (142/ 165)	156 (73/ 83)	–	42%	82.9%	68.9%	61.3%
**D. Y. Lim. et al**. [72]	Logistic Regression	12,117 (100/ 12,017)	5,193 (43/ 5,150)	0.815 (0.745–0.885)	–	–	–	–
	GLMNet	12,117 (100/ 12,017)	5,193 (43/ 5,150)	0.864 (0.804–0.924)	–	–	–	–
	Random Forests	12,117 (100/ 12,017)	5,193 (43/ 5,150)	0.826 (0.756–0.897)	–	–	–	–

* indicates that the result is from external validation data.

Abbreviations: LVH, Left Ventricular Hypertrophy; AUROC, area under the receiver operating characteristic curve operating characteristics curve; CI, confidence interval; PPV, positive predictive value; NPV, negative predictive value; CHCM, cardiac hypertrophy computer-based model; GLM, generalized linear model

We compared the model results by sequentially reducing the input to determine whether ECG has predictive power in diagnosing LVH. The model that learned the entire dataset showed a rapid decrease in performance when the ECG features were excluded from the input. It was possible to confirm the diagnostic tendency based on the characteristic data rather than the diagnostic power of the ECG itself. The model with the female dataset showed low diagnostic power overall, regardless of the input. However, the model with the male dataset showed no significant differences in performance. Nonetheless, the models with 6- and single-lead ECGs showed slightly reduced performance. It was confirmed that the ECG had LVH diagnostic power in male subjects. Furthermore, there was no significant difference in NPV between the model with the single-lead ECG and the model with 12-lead ECGs (97.87% vs. 97.12% and 96.54%, respectively). Therefore, we could consider the possibility of its application to wearable devices that have been used recently.

Our study has a few limitations. First, there is a discrepancy: the cut-off we used was a Western standard, and the dataset consisted of Asian races. ECG has slightly different morphological characteristics depending on the race [73]. A recent study reported that ECG voltage criteria verified through Westerners are not applicable to Asians and emphasized the application of voltage criteria considering race [74]. As such, ECG is influenced not only by gender but also by race, which is an essential consideration when analyzing ECG using deep learning [75]. Our study considered sex but not race, and this discrepancy may have affected the outcome. Attempts are needed to reduce this discrepancy through further studies, such as selecting an optimal cut-off appropriate for Asian races with LVH. Second, this study requires external validation. Many DL models have shown promising results in the verification process. However, it is difficult to demonstrate the generalization performance in a dataset environment with a slightly changed environment. Our model is no exception, because our data are a source dataset extracted from one hospital. Although there is also the problem mentioned above regarding racial bias, there may be other problems, such as regional bias and measuring device bias; therefore, verifying generalization capabilities using external datasets is necessary. Third, the data were collected from tertiary hospitals. Subjects who underwent ECG at tertiary hospitals were likely to have certain diseases or risk factors in advance. These dictionary elements can affect the ECG signals used in the experiment. This could be a potential cause of misclassification in the diagnosis of LVH. In particular, these potential effects may be even more remarkable because the subjects used in the experiment were those who underwent both ECG and echocardiography. Finally, the explicability of the models is insufficient. The model derives simple results, such as decisions, predictions, and diagnoses. Conversely, there is a limitation in that it is not possible to logically explain the validity and reliability of these final results, such as the basis or the process of deriving them. Thus, several explainable AI technologies have been developed, and we used Grad-CAM analysis. We checked the basis for our model’s determination in the diagnosis of LVH, and confirmed that it focuses on the QRS complex. However, there is a lack of a direct basis for wave characteristics that are viewed and predicted. It is necessary to confirm whether the demographic and ECG features used together affect it. Therefore, further research is required to ensure further explicability.

If additional data for external validation is obtained through future work of this study, the performance verification of the model will be performed again. In addition, we plan to use an additional model analysis method other than Grad-CAM to determine which characteristics and parts of the ECG were used for LVH diagnosis.

## Conclusion

In summary, this study developed a DL algorithm that can diagnose LVH using large-scale ECG big data. Our research makes the following contributions: First, to the best of our knowledge, we utilized a dataset containing the largest number of subjects among studies diagnosing LVH using ECG. The application of big data and DL showed that it overcame the limitations of existing research and achieved relatively better generalization performance. Secondly, we introduce a new ECG processing DL model, the CoAt-Mixer. We propose a more effective LVH diagnostic model based on self-attention through a framework that reflects ECG and demographic features. Finally, this study conducted a gender-conscious study. The difference in the heart between sexes was confirmed through previous studies. Therefore, the difference in diagnostic power between genders was identified through separate datasets, and the need for gender-consideration studies was emphasized.

The summary of abbreviations and acronyms used in the study and supporting information are summarized as follows (Table 5).

**Table 5:** Acronym table.

Abbreviation	Meaning
AUROC	area under the receiver operating characteristic
BSA	body surface area
CHCM	cardiac hypertrophy computer-based model
CI	confidence interval
CIs	confidence intervals
CNN	convolutional neural network
CT	computed tomography
DL	deep learning
ECG	electrocardiography
EF	ejection fraction
FN	false negative
FNN	feedforward neural network
FP	false positive
GeLU	gaussian error linear unit
GLM	generalized linear model
Grad-CAM	gradient-weighted class activation
J	youden index
LA	left atrium
LV	left ventricular
LVH	left ventricular hypertrophy
LVMI	left ventricular mass index
ML	machine learning
MRI	magnetic resonance imaging
NPV	negative predictive value
PPV	positive predictive value
ReLU	rectified learning unit
RNN	recurrent neural network
ROC	receiver operating characteristic
SD	standard deviation
TN	true negative
TP	true positive

The table describes the meaning of various abbreviations and acronyms used throughout this study.

## Supporting information

S1 Table Performance metrics for classification task with 10-fold cross validation. (DOCX)
