# Supplementary material for: CoAt-Mixer: Self-attention deep learning framework for left ventricular hypertrophy using electrocardiography
Source: PLoS One. 2023 Jun 8;18(6):e0286916. doi: 10.1371/journal.pone.0286916 (PMC10249819; doi:10.1371/journal.pone.0286916)
Supplement: S1 Table — (DOCX) [file pone.0286916.s001.docx]

**S1 Table. Performance metrics for classification task with 10-fold cross validation.**

| Gender | Input Data | Metrics | | | | | |
| --- | --- | --- | --- | --- | --- | --- | --- |
|  |  | **AUROC (95% CI)** | **Sensitivity (95% CI)** | **Specificity (95% CI)** | **PPV (95% CI)** | **NPV (95% CI)** | **F1 score (95% CI)** |
| Entire | 12-Lead ECGs  Demographics, ECG features | 0.833 (0.813 – 0.852) | 80.50% (75.10 – 85.91) | 71.05% (68.02 – 74.09) | 31.67% (30.31 – 33.03) | 95.72% (94.74 – 96.70) | 45.33% (43.78 – 46.89) |
| Male | 12-Lead ECGs  Demographics, ECG features | 0.840 (0.828 – 0.852) | 74.36% (70.29 – 78.44) | 78.16% (74.11 – 82.21) | 19.69% (17.35 – 22.03) | 97.79% (97.54 – 98.05) | 30.91% (28.20 – 33.61) |
| Female | 12-Lead ECGs  Demographics, ECG features | 0.791 (0.783 – 0.799) | 73.79% (69.52 – 78.06) | 69.09% (65.50 – 72.69) | 43.05% (41.30 – 44.81) | 89.48% (88.46 – 90.50) | 54.17% (53.42 – 54.93) |

Abbreviations: AUROC, area under the receiver operating characteristic curve; CI, confidence interval; PPV, positive predictive value; NPV, negative predictive value
